# L‐EasyARG: A long‐read metagenomics tool for rapid antibiotic‐resistance‐gene profiling

**DOI:** 10.1002/imo2.70098

**Published:** 2026-04-21

**Authors:** Yongxin Li, Mingchao Wang, Xiaohui Liu, Junhua Wu, Yujie Mao, Caili Zhang, Qingru Chen, Zhi Wang, Gongchao Jing, Maozhen Han

**Affiliations:** ^1^ School of Life Sciences Anhui Medical University Hefei China; ^2^ Microbial Medicinal Resources Development Research Team Anhui Provincial Institute of Translational Medicine Hefei China; ^3^ Qingdao University of Science and Technology Qingdao China; ^4^ Single‐Cell Center, CAS Key Laboratory of Biofuels and Shandong Key Laboratory of Energy Genetics, Qingdao Institute of BioEnergy and Bioprocess Technology Chinese Academy of Sciences Qingdao China; ^5^ Key Laboratory of Marine Environment and Ecology, Ministry of Education and College of Environmental Science and Engineering Ocean University of China Qingdao China; ^6^ Key Laboratory for Environment and Disaster Monitoring and Evaluation of Hubei, Innovation Academy for Precision Measurement Science and Technology Chinese Academy of Sciences Wuhan China; ^7^ Key Laboratory of Evolution & Marine Biodiversity (Ministry of Education) and Institute of Evolution & Marine Biodiversity Ocean University of China Qingdao China

## Abstract

l‐EasyARG is a pipeline for long‐read metagenomic profiling of antibiotic resistance genes (ARGs) and their genetic context. It integrates ARG identification, plasmid and mobile genetic element (MGE) detection, taxonomic classification, and further identifies ARGs carried by human bacterial pathogens (HBPs) defined in the WHO and ESKAPE pathogen lists. Benchmarking on simulated datasets with error rates ranging from 0% to 15% demonstrates optimal F1‐scores at ≤5% errors, outperforming existing tools such as ARGpore2 and DeepARG. Application to hospital wastewater samples validates its ability to capture disinfection‐induced shifts in ARG composition, ARG‐MGE co‐occurrence, and ARG‐host associations. l‐EasyARG generates comprehensive outputs including abundance profiles, co‐occurrence networks, and interactive visualizations, enabling efficient resistome surveillance and risk assessment. The pipeline is user‐friendly and locally executable.

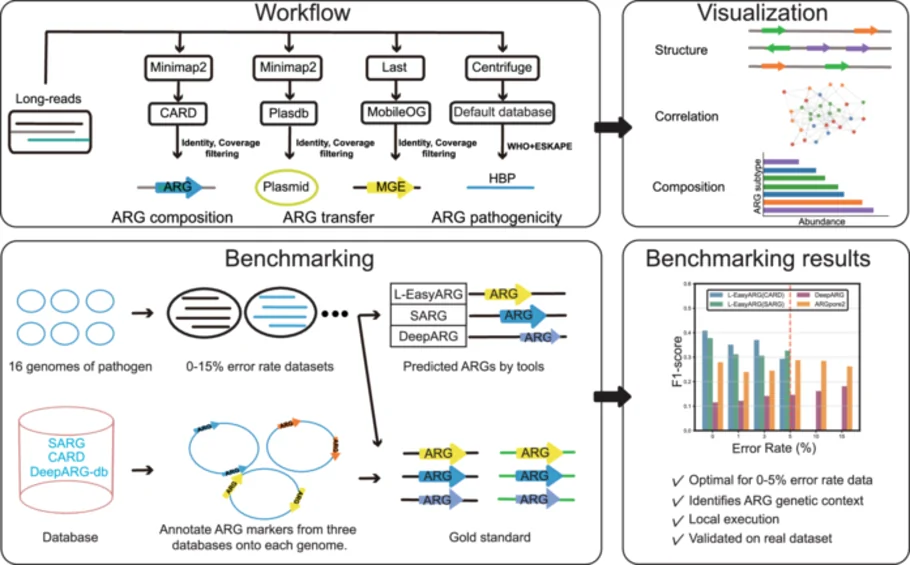

To the Editor,

Antimicrobial resistance (AMR), which enables bacteria to survive antibiotic exposure, has been recognized by the World Health Organization (WHO) as one of the foremost global health threats [1]. The widespread use of antibiotics has increased their concentrations in the environment, putting selective pressure on bacteria to acquire antibiotic resistance genes (ARGs) and potentially fostering the emergence of multidrug‐resistant pathogens [2], which poses a significant risk to public health. Once bacteria have acquired ARGs, they can disseminate them not only vertically through replication, but also horizontally to other bacteria via mechanisms such as horizontal gene transfer (HGT) [3]. For instance, ARGs are often carried on plasmids, which can transpose and integrate into new genomic locations. Evidence has indicated that microbial communities and mobile genetic elements (MGEs), particularly plasmids, are key drivers in the distribution and propagation of ARGs [2]. By framing AMR as a threat shared by humans, animals, and the environment, the “One Health” concept underscores the crucial need to research ARGs and their genetic contexts across diverse environmental habitats. However, the short‐read length results in fragmented metagenomic assemblies, making it difficult to link ARGs to their hosts [4].

The emergence and advancement of long‐read sequencing technologies, such as Nanopore and PacBio, help mitigate issues like fragmented contigs and assembly errors by generating significantly longer reads. These characteristics make long‐read sequencing particularly valuable for investigating the distribution of environmental ARGs and their genomic transmission mechanism. It should be noted that although long‐read sequencing has been applied to profile the ARGs and track their hosts, unassembled long‐read data, due to their elevated error rates, cannot follow the short‐read strategy of first calling open reading frames on assembled contigs and then querying databases. Instead, ARG identification currently relies on direct best‐match classifiers exemplified by ARGpore2 [5], l‐ARRAP [6], and NanoOK RT [7], or on machine‐learning models such as DeepARG [8].

Although these tools are capable of detecting ARGs in nanopore datasets, most offer limited capabilities for the comprehensive characterization of the genetic context of these genes. Recently, TELSeq and its companion pipeline TELCoMB [9] demonstrated that target‐enriched long‐read sequencing effectively resolves ARG‐MGE co‐localizations. However, these approaches require wet‐lab probe‐based enrichment, limiting their applicability to specialized laboratories. While NanoARG [10] provides detailed contextual analysis, its utility is hampered by a less frequently updated database and the lack of a local version, restricting its practical application. Furthermore, there are several key aspects of applying nanopore sequencing to environmental resistome studies that have yet to be adequately addressed. These include determining optimal ARG similarity thresholds, comprehensively benchmarking the performance of different tools, and systematically evaluating the impact of sequencing error rates on identification accuracy. To address these gaps, we developed l‐EasyARG, a tool that not only identifies ARGs effectively but also provides comprehensive genetic context analysis and visualization capabilities. Moreover, a systematic benchmark analysis of prevailing ARG identification tools was conducted in order to establish practical guidelines for the field.

## 1 RESULTS AND DISCUSSION

### Overview of the L‐EasyARG workflow

Both FASTQ and FASTA formats are supported. It adopts the best‐hit approach for the identification of ARGs, MGEs, and Plasmids. Here, “best‐hit” means that for each sequence, if multiple matches from the same database overlap by 80% or more in length, only the single match with the highest nucleotide identity is retained. The workflow begins by aligning reads against the Comprehensive Antibiotic Resistance Database (CARD) using Minimap2. Putative ARGs are identified from alignments meeting minimum thresholds of 60% for both identity and coverage, a combination determined to yield the highest F1‐score for ARG identification in our benchmarking analysis. Subsequently, to assess mobilization potential, L‐EasyARG uses the Minimap2 and LAST tools to align the reads to the Plasmid Database (PLSDB) and mobile orthologous groups (MobileOG) database, respectively. An ARG is considered to have potential mobility if it is found on the same long read as sequences aligned to plasmids or MGEs. This co‐occurrence suggests genetic linkage and potential horizontal transfer risk.

For taxonomic classification, l‐EasyARG employs the Centrifuge classifier, which is more suitable for long reads, and annotates the reads using its default parameters and built‐in database. Reads classified as species listed in the WHO or ESKAPE databases (*Enterococcus faecium*, *Staphylococcus aureus*, *Klebsiella pneumoniae*, *Acinetobacter baumannii*, *Pseudomonas aeruginosa*, *Enterobacter species*) are designated as human bacterial pathogens (HBPs) (Table S1). The pathogenic potential of an ARG is then assessed based on its co‐occurrence with a HBP classification on the same read. Overall, L‐EasyARG is designed to profile three key aspects of ARGs: their composition, mobilization potential, and pathogenic potential. The complete workflow is depicted in Figure 1A, with detailed methods provided in the Supplementary Methods.

**Figure 1:**
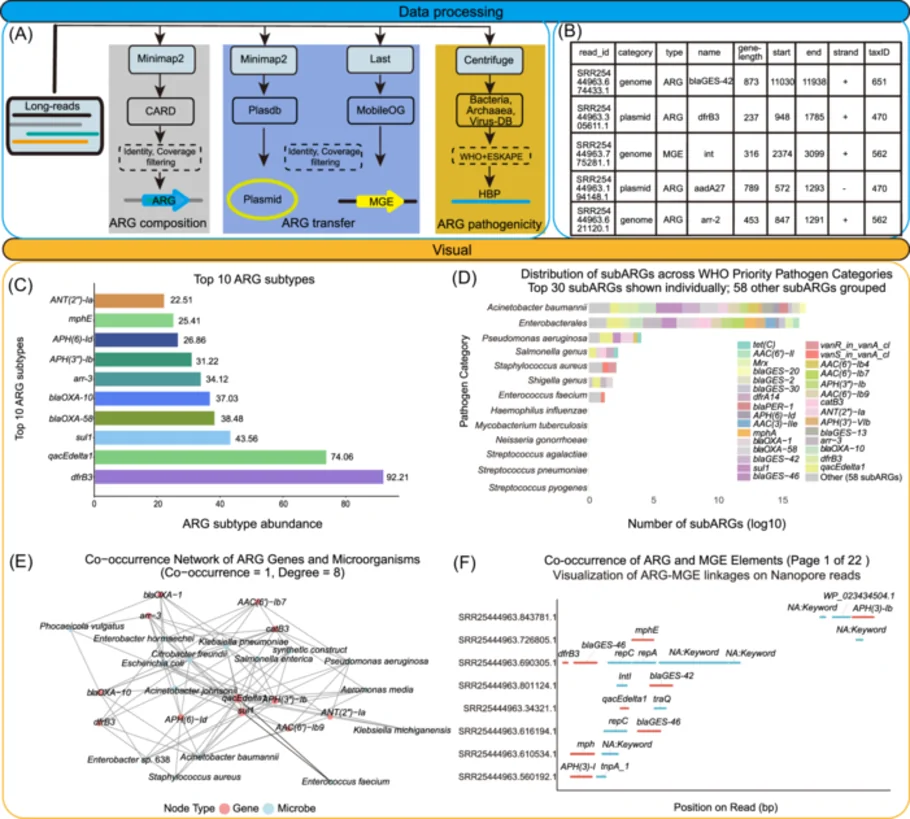
Workflow of L‐EasyARG including ARGs identification, MGE identification, plasmid identification, taxonomic annotation, statistical analysis, and visualization. (A) Workflow of L‐EasyARG. (B) Catalog of all ARG‐containing long reads, including their genomic positions, strand orientation, and taxonomic assignments to enable analysis of co‐localization with MGEs and HBPs. (C) Bar chart showing the abundance of the top 10 ARG subtypes across all sequencing reads. (D) Stacked bar chart showing the distribution of ARG subtypes across HBPs. (E) Co‐occurrence network depicting the associations between ARGs and HBPs. (F) Genetic co‐localization of ARGs and MGEs on long‐read sequences. Red and blue bars represent ARGs and MGEs, respectively. ARG, antibiotic resistance gene; CARD, Comprehensive Antibiotic Resistance Database; ESKAPE, *Enterococcus faecium*, *Staphylococcus aureus*, *Klebsiella pneumoniae*, *Acinetobacter baumannii*, *Pseudomonas aeruginosa*, *Enterobacter species*; HBP, human bacterial pathogen; MGE, mobile genetic element; PLSDB, Plasmid Database; MobileOG, mobile orthologous groups; subARG, subtype of ARG; taxID, taxonomy ID.

The primary output is a core results table that catalogs all ARG‐containing reads and their genetic contexts (Figure 1B). Each entry in the table contains more detailed information, including the start position, end position, strand (forward or reverse), and the corresponding taxonomic classification for each read. This comprehensive table subsequently underpins the automated generation of a suite of interactive visualizations implemented in Python and R scripts. These include profiles of the total ARG copy number and the ARG copy number carried by HBPs (Figure 1C,D), an ARG‐MGE co‐occurrence map (Figure 1E), and an ARG‐HBP co‐occurrence network (Figure 1F), collectively providing an intuitive and comprehensive resistome profile.

### Benchmarking analysis

To determine the optimal parameters for l‐EasyARG and objectively benchmark its performance against existing tools, we conducted a systematic evaluation. First, 16 complete pathogenic bacterial genomes (Table S2) were downloaded from NCBI. From these, we constructed a series of benchmark datasets by simulating long‐read sequencing data at 6× coverage per genome across a gradient of sequencing error rates (0%, 1%, 3%, 5%, 10%, and 15%) using Badread [11]. Detailed simulation parameters and dataset construction are provided in Supplementary Methods.

To establish an accurate ground standard for evaluation, gene annotations and genomic coordinates for these 16 genomes were obtained from NCBI. We then constructed tool‐specific gold standard annotations by identifying true ARGs via BLAST alignment of the annotated gene sequences against each tool's respective reference database (CARD, SARG, or DeepARG‐db) and mapped their coordinates to the simulated reads (Supplementary Methods). Next, using the error‐free (0%) dataset, we performed a grid search to determine the optimal identity and coverage thresholds for Minimap2 alignment within L‐EasyARG (Supplementary Methods). A prediction was considered a true positive only if Minimap2 identified the exact same ARG subtype within the same read as specified in the gold standard annotation. The highest accuracy (F1‐score) for ARG identification was achieved when both thresholds were set to 0.6 (Figure 2A), and this combination was consequently adopted as the default (detailed formulas in Supplementary Methods).

**Figure 2:**
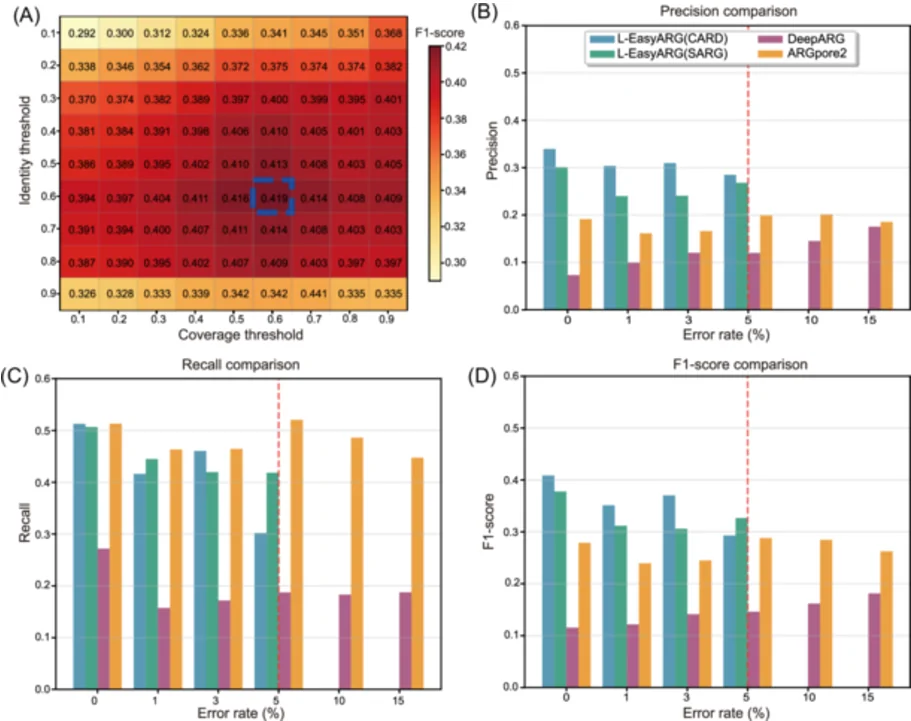
Benchmark analysis results. (A) F1‐scores of Minimap2 under varying identity and coverage filtering thresholds. The optimal threshold was 0.6 for both identity and coverage. The blue dashed box highlights the combination of identity and coverage thresholds (both 0.6) that achieved the highest F1‐score, selected as the default parameters for L‐EasyARG. Precision (B), Recall (C), and F1‐scores (D) of L‐EasyARG, DeepARG, and ARGpore2 across varying error rates. The red dashed line in panels B‐D marks the 5% sequencing error rate, corresponding to the upper bound of typical error rates for recent Nanopore chemistry (e.g., R10.4 flow cells). In our case study, the estimated error rates ranged from 2.57% to 4.51% (see Table S3), falling within this range where L‐EasyARG maintains optimal performance.

We then benchmarked l‐EasyARG against two popular long‐read ARG profiling tools, ARGpore2 and DeepARG, which cover different analytical approaches (NanoARG was excluded because it employs a methodology similar to DeepARG and requires data upload). To ensure a fair comparison, tool‐specific gold standard annotations were constructed based on their respective reference databases (CARD, SARG, and DeepARG‐db), and all tools were evaluated across the same set of six error‐rate datasets (Supplementary Methods). Tool performance was assessed using three standard metrics: Precision (the fraction of correct predictions among all positive calls), Recall (the fraction of true ARGs successfully detected), and their harmonic mean, the F1‐score (detailed formulas in Supplementary Methods).

The performance evaluation demonstrated that L‐EasyARG achieved the highest accuracy and F1‐score across all low error‐rate conditions (0%, 1%, 3%, and 5%), benefiting from Minimap2's efficient seeding strategy under high accuracy. In contrast, ARGpore2, leveraging LAST's frameshift‐sensitive mode, demonstrated consistent stability across all tested error rates, exhibiting the best performance when the error rate exceeded 5%. DeepARG consistently underperformed because its reliance on a reference‐based dissimilarity model rendered input features vulnerable to sequencing errors. A detailed analysis of these algorithmic distinctions is provided in Supplementary Methods. To further validate the performance of L‐EasyARG, we performed an additional benchmarking analysis where L‐EasyARG was configured to use the SARG database instead of CARD. The results confirmed that L‐EasyARG, whether using the CARD or SARG database, consistently yielded higher F1‐scores than both ARGpore2 and DeepARG at error rates below 5%. To assess the practical data quality in real‐world scenarios, we determined the error rates of the eight hospital wastewater samples used in our subsequent application study (Supplementary Methods). The error rates ranged from 2.57% to 4.51% (Table S3), which falls within the optimal performance range of L‐EasyARG. It is noteworthy that with the continuous advancement of Nanopore sequencing technology, the latest R10.4 version can control the error rate to below 2% [12], highlighting the significant application potential of L‐EasyARG in practical scenarios (Figure 2B–D).

## 2 CONCLUSION

Benchmarking on simulated data established the accuracy of L‐EasyARG under realistic sequencing error rates. To further demonstrate its functionality, we applied it to a hospital wastewater dataset (BioProject: PRJNA999488) [13]. The tool confirmed the key shifts in the resistome reported after disinfection, including increased ARG abundance, increased mobilization potential of ARGs, and increased abundance of ARGs carried by HBPs. A detailed presentation of all results, including ARG compositional shifts, β‐diversity analysis (PCoA), ARG‐MGE co‐occurrence, and statistical comparisons, is provided as a case study in Figure S1.

This application validates that l‐EasyARG is not merely a detection tool but an integrated pipeline specifically designed for long‐read data. By combining ARG and MGE identification, taxonomic annotation, and pathogen screening into a user‐friendly, locally executable workflow, it makes comprehensive resistome profiling accessible to researchers with varying levels of computational expertise. In summary, L‐EasyARG provides an efficient, standardized workflow for analyzing ARGs in complex environmental metagenomes, demonstrating strong potential for monitoring the dissemination and ecological contexts of antibiotic resistance across diverse settings.

While L‐EasyARG demonstrates high accuracy and utility within its optimal operating range, users should be aware of its limitations to ensure appropriate application. Sequencing Error Rate Dependency: The performance of L‐EasyARG, which relies on Minimap2 for sequence alignment, is highly dependent on read accuracy. Our benchmarking shows optimal performance (highest F1‐score) at error rates below 5%, with a rapid decline in sensitivity beyond 10%. Therefore, we strongly recommend applying it to long‐read data with an estimated error rate of ≤5%. For Oxford Nanopore sequencing, this typically corresponds to data generated using R10.3 or later flow cells, which routinely achieve error rates below 5%. Analysis of data from older chemistries (e.g., R9.4) may require prior quality assessment and filtering.

## AUTHOR CONTRIBUTIONS

**Yongxin Li**: Software; formal analysis; writing—original draft. **Mingchao Wang**: Software; writing—original draft; writing—review and editing. **Xiaohui Liu**: Writing— original draft; writing—review and editing. **Junhua Wu**: Writing—Review and editing. **Yujie Mao**: Writing—review and editing. **Caili Zhang**: Writing—review and editing. **Qingru Chen**: Writing—review and editing. **Zhi Wang**: Conceptualization. **Gongchao Jing:** Conceptualization. **Maozhen Han**: Conceptualization; data Curation. All authors have read the final manuscript and approved it for publication.

## CONFLICT OF INTEREST STATEMENT

The authors declare no conflicts of interest.

## ETHICS STATEMENT

No animals or humans were involved in this study.

## AI STATEMENT

During the preparation of this work, we used DeepSeek (version DeepSeek‐R1) to polish the initial draft of the manuscript. After using this tool, we reviewed and edited the content as needed and took full responsibility for the final content of the publication.

## Supporting information


**Figure S1.** Analysis of antibiotic resistome dynamics in hospital wastewater following disinfection using L‐EasyARG.

**Table S1.** The important drug‐resistant pathogens from WHO and ESKAPE.
**Table S2.** Lists the 16 pathogenic bacterial genomes that were simulated using Badread and subsequently used for the benchmark analysis of the tools.
**Table S3.** The detailed information of HWW samples used in this study.

## Data Availability

All L‐EasyARG code is available on GitHub (https://github.com/liyonxin/l‐EasyARG). Public dataset PRJNA999488, comprising hospital wastewater samples, was downloaded from the NCBI Sequence Read Archive (SRA) for use in this study. Supplementary materials (figures, tables, methods, graphical abstract, slides, videos, Chinese translated version, and updated materials) may be found in the online DOI or iMetaOmics Science http://www.imeta.science/imetaomics.
