# Supplementary material for: L‐EasyARG: A long‐read metagenomics tool for rapid antibiotic‐resistance‐gene profiling
Source: IMetaOmics. 2026 Apr 21;3(2):e70098. doi: 10.1002/imo2.70098 (PMC13674386; doi:10.1002/imo2.70098)
Supplement: Supplementary file 1 [file IMO2-3-e70098-s001.docx]

Supporting Information to

**L-EasyARG: a long-read metagenomics tool for rapid antibiotic-resistance-gene profiling**

**Running title: L-EasyARG for long-read ARG detection**

Yongxin Li^1, 2#^, Mingchao Wang^3, 4#^, Xiaohui Liu^5#^, Junhua Wu^1^, Yujie Mao^6^, Caili Zhang^1, 2^, Qingru Chen^1, 2^, Zhi Wang^6 *^, Gongchao Jing^7*^, Maozhen Han^1, 2*^

^1^ School of Life Sciences, Anhui Medical University, Hefei 230032, China

^2^ Microbial medicinal resources development research team, Anhui Provincial Institute of Translational Medicine, Hefei 230032, China

^3^ Qingdao University of Science and Technology, Qingdao 266000, China

^4^ Single-Cell Center, CAS Key Laboratory of Biofuels and Shandong Key Laboratory of Energy Genetics, Qingdao Institute of BioEnergy and Bioprocess Technology, Chinese Academy of Sciences, Qingdao 266100, China

^5^ Key Laboratory of Marine Environment and Ecology, Ministry of Education and College of Environmental Science and Engineering, Ocean University of China, Qingdao 266100, China

^6^ Key Laboratory for Environment and Disaster Monitoring and Evaluation of Hubei, Innovation Academy for Precision Measurement Science and Technology, Chinese Academy of Sciences, Wuhan 430077, China

^7^ Key Laboratory of Evolution & Marine Biodiversity (Ministry of Education) and Institute of Evolution & Marine Biodiversity, Ocean University of China, Qingdao 266003, China

^#^ These authors contributed equally: Yongxin Li, Mingchao Wang, Xiaohui Liu

^*^Correspondence: [hanmz@ahmu.edu.cn](mailto:hanmz@ahmu.edu.cn) (Maozhen Han), [jinggc@qibebt.ac.cn](mailto:jinggc@qibebt.ac.cn) (Gongchao Jing), [zwang@apm.ac.cn](mailto:zwang@apm.ac.cn) (Zhi Wang)

**METHODS**

**Identification of ARGs, MGEs, and plasmids**

L-EasyARG employs a best-hit approach for identifying ARGs, MGEs, and plasmids. The pipeline first aligns reads against the Comprehensive Antibiotic Resistance Database (CARD) nucleotide sequences [1] using Minimap2 (v2.30) [2]. The alignment is performed with the “*-x*” preset selected according to the sequencing technology (*e.g.*, map-ont for Oxford Nanopore data) and the “*--secondary=no*” flag to report only primary alignments, minimizing ambiguous mappings.

As MGEs are able to capture ARGs from chromosomes and transfer them horizontally to other bacteria via plasmids or phages [3], special attention should be paid to MGEs when assessing the antibiotic risk. Similar to the ARG annotation strategy, long-read sequences were aligned to the PLSDB database (v2) [4] by Minimap2 (v2.30) to determine whether the detected ARGs are carried by plasmids. The default identity and coverage thresholds are both 0.6, which is consistent with the method of ARG identification. This harmonization ensures a consistent standard for defining positive hits across different modules of the pipeline, facilitating the integrated analysis of ARG-plasmid associations.

For profiling MGEs, reads are aligned to the protein sequences of the MobileOG-db (v1.6) [5] using LAST [6] (v.1645) with parameters “*-p -m100 -D1e9 -K1*” to ensure high-confidence protein-level matches. The alignment process is preceded by database indexing (*lastdb*) and parameter optimization (*last-train*) steps tailored to the input reads. The default cut-off value of identity and coverage threshold is 0.7/0.75, which is consistent with the method of ARGpore2 [7]. Only the ARG/MGE hits of the highest mapping quality are kept whenever hits overlapped other ARG/MGE hits by ≥ 80% to avoid multiple hits on the same gene segment.

According to the calculation method described by *Ma et al*. [8], the abundances of ARG and MGE are calculated as the number of copies of the ARG/MGE per gigabyte of base pairs:

$$\text{Abundance}_{\text{ARG/MGE}}\text{(coverage, ×/Gb)=}\frac{\text{N}_{\text{mapped genes}}}{\text{S}}$$

Where *N* is the total number of reference gene(s) for ARGs or MGEs; and *S* represents the total sequencing data size in Gigabases (Gb), which is calculated by dividing the total number of sequenced bases (in base pairs) from all reads in a given sample by 10^9^.

**Taxid classification**

In this study, the long reads that contain ARGs were taxonomically classified using the Centrifuge tool [9]. To further assess the clinical risk associated with the identified ARGs, L-EasyARG further cross-references the results with two critical databases of clinically relevant pathogens. Specifically, the classified pathogens are compared against a list of priority pathogens published by the WHO (proposed in 2024) [10], which categorizes pathogens into critical, high, and medium priority tiers based on their urgency for research and development of new antibiotics of new antibiotics. Additionally, comparison is made against the ESKAPE (proposed in 2013) [11] database, which encompasses the major multidrug-resistant bacterial pathogens (*Enterococcus faecium, Staphylococcus aureus, Klebsiella pneumoniae, Acinetobacter baumannii, Pseudomonas aeruginosa, and Enterobacter spp.*) that pose a severe threat to human health. By determining which ARGs are located in these HBPs, L-EasyARG provides crucial insights for evaluating the potential public health risk and clinical impact of the detected antibiotic resistance. The abundance for each pathogen belonging HBPs was calculated as follows:

$$\text{Abundance}_{\text{HBP}}\text{(coverage, ×/Gb)=}\frac{\text{N}_{\text{mapped reads}}}{\text{S}}$$

Where *N* is the total number of reads for HBPs; and *S* represents the total sequencing data size in Gigabases (Gb), which is calculated by dividing the total number of sequenced bases (in base pairs) from all reads in a given sample by 10^9^.

**Comparison with existing long-read ARG context workflows**

L-EasyARG was compared with TELSeq/TELCoMB pipelines. While TELSeq employs target-enriched long-read sequencing combined with TELCoMB analysis (requiring wet-lab biotinylated probe capture), L-EasyARG operates as a purely bioinformatics pipeline on standard long-read data. Key technical distinctions include: (1) L-EasyARG uses CARD (nucleotide) for ARGs and MobileOG (protein) for MGEs, whereas TELCoMB uses MEGARes and ACLAME/ICEberg; (2) L-EasyARG employs systematically benchmarked thresholds (60% identity/60% coverage for ARGs, 70%/75% for MGEs) to maximize F1-scores under typical nanopore error rates (2.5–4.5%), compared to TELSeq’s default 80% gene fraction cutoff; (3) L-EasyARG defines mobilization potential through co-occurrence on single reads with plasmids (PLSDB) or MGEs (MobileOG), coupled with taxonomic host assignment via Centrifuge and pathogenic potential scoring via WHO/ESKAPE databases, whereas TELCoMB focuses primarily on ARG-MGE colocalization without host-pathogen risk stratification; (4) L-EasyARG’s benchmarking explicitly evaluates performance across 0–15% error rates, demonstrating optimal accuracy below 5%, which is critical for real-world nanopore applications.

**Benchmark dataset construction**

Sixteen complete genomes of pathogenic bacteria (Table S2) were downloaded from the NCBI database. Long reads were simulated from each genome using Badread (v0.4.1) to mimic Nanopore sequencing data. The simulation was performed with the following core parameters for all genomes: --quantity *6x* (coverage), *--length 15000,5000* (mean length 15,000 bp, standard deviation 5,000 bp), *--error_model nanopore2023*, *--qscore_model nanopore2023*, and with glitches, chimeras, random reads, and junk reads disabled (*--glitches 0,0,0 --chimeras 0 --random_reads 0 --junk_reads 0*) to isolate the effect of nucleotide substitution errors on tool performance, excluding confounding factors from complex sequencing artefacts. The primary substitution error rate was controlled via the --identity parameter. A perfect, error-free dataset (0% error rate) was generated using *--identity 100,100,100*. Datasets with approximate error rates of 1%, 3%, 5%, 10%, and 15% were subsequently generated by setting *--identity* to *99,99,99*, *97,97,97*, *95,95,95*, *90,90,90*, and *85,85,85*, respectively.

To generate a standardized and reproducible test set for benchmarking, 10,000 reads were randomly subsampled from the pooled reads using *seqtk sample -s 100* (v1.4) [12]. The fixed random seed (*-s 100*) ensures the exact same subset can be obtained for reproducible evaluation. This process resulted in six distinct benchmark test datasets (one per error rate), each containing 10,000 reads.

To ensure a fair and accurate evaluation, we constructed separate gold standard-annotations for each benchmarking tool (L-EasyARG, ARGpore2, and DeepARG), accounting for their respective underlying reference databases.

**Database-specific ARG annotation:** The protein-coding gene sequences from the 16 genomes were aligned against the primary database used by each tool: the CARD database (for L-EasyARG), the SARG database (for ARGpore2), and the DeepARG database (for DeepARG), using BLAST with a stringent threshold (≥ 97% amino acid identity and coverage). Genes meeting this threshold for a given database were defined as the “true ARGs” for the corresponding tool.

**Read-level gold standard mapping:** The genomic coordinates of these database-specific “true ARGs” were mapped to the simulated reads in each of the six error-rate datasets (10,000 reads per set). An ARG was considered “present” in a read if their genomic intervals overlapped. This process generated unique gold standard annotation file for each combination of tool (database) and error-rate dataset.

All tools were evaluated on the six error-rate datasets using their default or recommended parameters to ensure a fair and unbiased comparison. The predictions from each tool on a given dataset were then compared exclusively to its corresponding tool-specific gold standard for that error rate. Following the evaluation methodology of *Wu et al*. [7], a prediction was counted as a true positive (TP) only if the tool identified the exact same ARG subtype on the same read as annotated in its own gold standard.

**Determination of optimal alignment thresholds for L-EasyARG**

The optimal identity and coverage thresholds for Minimap2 alignment within the L-EasyARG pipeline were determined using the 0% error-rate benchmark dataset and its corresponding CARD-based gold standard. The test reads were aligned to the CARD database using Minimap2. The alignment results were then filtered using a systematic grid search across 81 distinct combinations of identity and coverage thresholds (each ranging from 0.1 to 0.9 in steps of 0.1). A prediction was considered a true positive only if Minimap2 identified the exact same ARG subtype within the same read as specified in the gold standard. Precision, Recall, and F1-score were calculated for each threshold combination. The combination that yielded the highest F1-score (The combination of 0.6 for both identity and coverage that yielded the highest F1-score) was selected as the default parameter for ARG recognition in L-EasyARG.

**Benchmarking evaluation metrics**

To objectively evaluate and compare the performance of ARG identification tools (L-EasyARG, ARGpore2, and DeepARG) under various sequencing error rates, we employed three standard classification metrics: Precision, Recall, and F1-score. These metrics were calculated based on the comparison between tool predictions and a tool-specific gold standard dataset. Their definitions, formulas, and interpretations in our context are as follows:

Precision quantifies the reliability of a tool’s positive predictions. It is defined as the proportion of correctly predicted ARG-containing reads (True Positives, TP) among all reads that the tool predicted to contain ARGs.

$$Precision=\frac{\mathrm{TP}}{TP+FP}$$

Note: A high Precision indicates that when the tool labels a read as containing an ARG, it is likely to be correct.

Recall quantifies a tool’s ability to detect all relevant ARGs present in the data. It is defined as the proportion of correctly predicted ARG-containing reads (TP) among all reads that actually contain ARGs according to the gold standard.

$$Recall=\frac{\mathrm{TP}}{TP+FN}$$

Note: A high Recall indicates that the tool successfully identifies most of the true ARG-positive reads (low false negative rate).

F1-score provides a single, balanced metric that harmonizes the trade-off between Precision and Recall. It is the harmonic mean of the two:

$$F1-score=2\times\frac{Precision\times Recall}{Precision+Recall}$$

Note: The F1-score is particularly useful when seeking a balance between minimizing false positives and false negatives. A higher F1-score indicates overall better accuracy.

**Definition and estimation of sequencing error rate**

The term “sequencing error rate” used in this study refers to the estimated per-base mismatch rate (incorporating both substitution and insertion/deletion errors) in the raw long-read sequences. For each Oxford Nanopore Technologies (ONT) sample, the mean Phred quality score (Q) was calculated using NanoPlot [13]. The per-base error rate (*E*) was then derived using the standard formula:

$$E={10}^{(-Q/10)}\times100\%$$

For example, a mean Q score of 20 corresponds to an estimated error rate of 1%. The error rates for all samples analyzed in this study are provided in Table S3**.**

**Statistical analysis**

All statistical analyses were performed using R software (version 4.5.2). Independent samples *t*-tests were conducted using functions from the base *stats* package. The significance level was set at *p* < 0.05. Since the tests for different metrics (*e.g.*, abundance of ARGs co-occurring with MGEs and abundance of ARGs carried by HBPs) addressed independent biological questions, no multiple comparison correction was applied.

Beta diversity analysis was based on the Bray-Curtis distance matrix and visualized using principal coordinate analysis (PCoA). Permutational multivariate analysis of variance (PERMANOVA) with 999 permutations was employed to test for differences in ARG composition between the two groups, with a significance threshold of *p* < 0.05. PERMANOVA was performed using the *vegan* package (version 2.7-2) in R. The correlation between ARG and HBP profiles was assessed using the Mantel test based on Pearson’s correlation coefficient, with 999 permutations.

**RESULTS**

**Discussion of benchmarking results**

The benchmarking results reveal distinct performance patterns for L-EasyARG, ARGpore2, and DeepARG across varying sequencing error rates. These differences are primarily attributable to their underlying alignment algorithms, database structures, and model training strategies.

L-EasyARG (Minimap2-based) excels at lower error rates (≤ 5%) due to its use of fixed-length minimizer seeding (default k = 15 for ONT reads). This approach efficiently identifies exact k-mer matches and constructs contiguous alignments when error rates are moderate [2]. However, as error rates exceed 5%, the probability of finding exact 15‑bp matches decreases significantly, leading to fragmented alignment chains and reduced sensitivity, especially for divergent ARG variants.

ARGpore2 (LAST-based) shows greater robustness at higher error rates (10–15%) because LAST employs an adaptive seed‑length strategy and a frameshift‑sensitive alignment mode optimized for Nanopore’s high indel rates (*e.g.*, homopolymer errors in R9.4.1 chemistry). By reducing gap penalties and allowing gapped alignments, LAST [6] maintains alignment continuity even under substantial sequencing noise, thereby sustaining higher recall at elevated error rates. This robustness, however, comes at increased computational cost compared to Minimap2.

DeepARG (DeepARG‑LS) exhibited lower overall performance on Nanopore‑simulated data because its deep‑learning model was trained on high‑accuracy reference sequences (from CARD, ARDB, and UNIPROT) without explicit adaptation to Nanopore error profiles. The model relies on bit‑score distributions from DIAMOND alignments as input features; high error rates distort these distributions, impairing its ability to recognize ARG‑like sequences. Furthermore, DeepARG is known to be less sensitive to single‑nucleotide polymorphisms (SNPs) and minor mutations, which are challenging to distinguish from sequencing errors in noisy long‑read data [14].

These observations underscore the importance of matching tool characteristics to data quality and research objectives when profiling ARGs from long‑read metagenomes.

**Implications for tool selection**

L-EasyARG is recommended for datasets with moderate error rates (≤ 5%), such as those generated with recent Nanopore chemistries (R10.3+) or after base‑calling improvements. ARGpore2 is more suitable for noisier data (*e.g.*, older R9.4.1 reads or samples with high intrinsic sequence diversity) where alignment continuity is critical. DeepARG‑LS may still be useful for short‑read metagenomes, but its performance on typical Nanopore data is limited.

**Performance evaluation of L-EasyARG**

To provide practical guidance for users, we evaluated the computational performance of L-EasyARG on a standard server (40 CPU threads, 128 GB RAM). Using a typical 1.3 GB nanopore sequencing dataset as input, the complete pipeline required approximately 1 h and 5 min of total wall-clock time to execute all analysis steps, including read classification and alignments against ARG, plasmid, and MGE databases. The process demonstrated efficient parallelization, with an average CPU utilization of 2806%. The peak memory usage was approximately 45.2 GB. Based on this benchmark, we recommend a minimum of 64 GB of RAM for reliably processing individual nanopore samples of comparable size (1–2 GB). Runtime and memory consumption are expected to scale approximately linearly with input data volume.

**Case study: Application of L-EasyARG in hospital wastewater samples**

**Dataset description:** The hospital wastewater (HWW) dataset analyzed in this study (BioProject: PRJNA999488) was obtained from the published work of *Rolbiecki et al*. [15], which investigated the impact of chlorine disinfection on the resistome and mobilome. Briefly, the dataset comprises eight samples: four collected before and four collected after the disinfection unit at a respiratory disease hospital in northeastern Poland, sampled seasonally from spring 2021 to winter 2022. The sole intervention between these paired conditions was chlorine-based disinfection, performed using an automated sodium hypochlorite system targeting a free chlorine concentration of 0.2 mg/L with a 1.5-hour contact time. Sample preprocessing involved filtration (0.2 μm) and DNA extraction using a commercial kit. Sequencing was conducted on Oxford Nanopore MinION devices (R10.4 flow cells). Complete methodological details are available in the source publication.

**Complete analysis results:** All analyses were performed using L-EasyARG with its default parameters. All abundance values are presented as the median with the range (minimum to maximum) across the four replicate samples within each group (pre- or post-disinfection) shown in parentheses.

**The composition of ARG:** Results of the L-EasyARG showed that disinfection increased the abundance of ARGs from 1133.9 ×/Gb to 2365.2 ×/Gb. Before disinfection, 141 different ARG subtypes were identified, while after disinfection, 284 different ARG subtypes were found. Additionally, the abundance of ARGs also significantly increased after disinfection. Before disinfection, the most abundant ARG subtypes were *blaOXA-58* (124.44 ×/Gb), *qacEdelta1* (93.13 ×/Gb), and *APH(3'')*-*Ib* (73.35 ×/Gb), while after disinfection, the most abundant subtypes were *dfrB3* (215.44 ×/Gb), *qacEdelta1* (193.49 ×/Gb), and *sul1* (147.43 ×/Gb) (Figure S1A). Beta diversity analysis is often combined with dimensionality reduction methods to assess differences in classification and structure among samples from different sources. Here, we used the PCoA method and found that the composition of ARGs before and after disinfection was significantly different; this result is consistent with the original study (Figure S1B) [15].

**ARG-MGE:** The main factors influencing the distribution of ARGs are widely recognized as MGEs, with plasmids being the primary driver of ARG dissemination. When ARGs co-occur with MGEs and plasmids, their potential for migration increases. Therefore, in the prevention and control of ARG pollution, the association between ARGs and MGEs as well as plasmids cannot be ignored. According to L-EasyARG analysis, disinfection significantly increased the abundance of ARGs co-occurring with MGEs from 305.2 (42.69–550.71) ×/Gb to 837.1 (535.83–1413.6) ×/Gb (t-test, *p* = 0.038) (Figure S1C). Interestingly, although the number of plasmid-borne ARG subtypes increased from 86 to 156 after disinfection, the abundance of plasmid-borne ARGs decreased from 586.9 (109.8–952.9) ×/Gb to 493.3 (355.1–717.6) ×/Gb (Figure S1D). Despite a slight decrease in the abundance of plasmid-borne ARGs, the significant increase in the abundance of ARGs co-occurring with MGEs after disinfection (*t*-test, *p* = 0.038) suggests that the increased mobilization potential of ARGs in mobile contexts after disinfection (Figure S1E).

**ARG-HBP:** A total of 28 pathogenic bacteria were detected both before and after disinfection, yet the total abundance significantly increased from 21930.67 ×/Gb to 40023.27 ×/Gb (*t*-test, *p* = 0.024). Specifically, 16 pathogenic species carrying 81 ARG subtypes were identified before disinfection, which increased to 26 species carrying 202 subtypes after disinfection. Furthermore, the abundance of ARGs hosted by HBPs significantly increased from 408.35 (54.89–639.04) ×/Gb to 1093.53 (750.02–1392.09) ×/Gb (*t*-test, *p* < 0.01). Notably, the dominant ARG subtype in HBP remained as *qacEdelta1* before and after disinfection, with its average abundance increasing from 42.45 ×/Gb to 75.67 ×/Gb (Figure S1F). A heatmap illustrating the correlations between the top five high-risk HBPs and the top 15 ARG subtypes is presented in Figure S1G. Collectively, these findings indicate a relative enrichment of ARGs within HBP-associated reads.

**REFERENCES**

1. Alcock, Brian P., Amogelang R Raphenya, Tammy T Y Lau, Kara K Tsang, Mégane Bouchard, Arman Edalatmand, et al. 2020. “CARD 2020: antibiotic resistome surveillance with the comprehensive antibiotic resistance database.” *Nucleic Acids Research* 48(D1): D517–D525. https://doi.org/10.1093/nar/gkz935

2. Li, Heng. 2018. “Minimap2: pairwise alignment for nucleotide sequences.” *Bioinformatics* 34(18): 3094–3100. https://doi.org/10.1093/bioinformatics/bty191

3. Ellabaan, Mostafa M H, Christian Munck, Andreas Porse, Lejla Imamovic, and Morten O A Sommer. 2021. “Forecasting the dissemination of antibiotic resistance genes across bacterial genomes.” *Nature Communications* 12(1): 2435. https://doi.org/10.1038/s41467-021-22757-1

4. Galata, Valentina, Tobias Fehlmann, Christina Backes, and Andreas Keller. 2019. “PLSDB: a resource of complete bacterial plasmids.” *Nucleic Acids Research* 47(D1): D195–D202. https://doi.org/10.1093/nar/gky1050

5. Brown, Connor L., James Mullet, Fadi Hindi, James E Stoll, Suraj Gupta, Minyoung Choi, et al. 2022. “mobileOG-db: A manually curated database of protein families mediating the life cycle of bacterial mobile genetic elements.” *Applied and Environmental Microbiology* 88(18): e00991–22. https://doi.org/10.1128/aem.00991-22

6. Kiełbasa, Szymon M., Raymond Wan, Kengo Sato, Paul Horton, and Martin C Frith. 2011. “Adaptive seeds tame genomic sequence comparison.” *Genome Research* 21(4): 487–493. https://doi.org/10.1101/gr.113985.110

7. Wu, Ziqi, You Che, Chenyuan Dang, Miao Zhang, Xuyang Zhang, Yuhong Sun, et al. 2022. “Nanopore-based long-read metagenomics uncover the resistome intrusion by antibiotic resistant bacteria from treated wastewater in receiving water body.” *Water Research* 226: 119282. https://doi.org/10.1016/j.watres.2022.119282

8. Ma, Liping, Yu Xia, Bing Li, Ying Yang, Li-Guan Li, James M Tiedje, et al. 2016. “Metagenomic assembly reveals hosts of antibiotic resistance genes and the shared resistome in pig, chicken, and human feces.” *Environmental Science & Technology* 50(1): 420–427. https://doi.org/10.1021/acs.est.5b03522

9. Song, Li, and Ben Langmead. 2024. “Centrifuger: lossless compression of microbial genomes for efficient and accurate metagenomic sequence classification.” *Genome Biology* 25(1): 106. [https://doi.org/10.1186/s13059-024-03244-4](https://doi.org/10.1186/s13059-024-03244-4)

10. Sati, Hatim, Elena Carrara, Alessia Savoldi, Paul Hansen, Jacopo Garlasco, Enrica Campagnaro, et al. 2025. “The WHO Bacterial Priority Pathogens List 2024: a prioritisation study to guide research, development, and public health strategies against antimicrobial resistance.” *The Lancet Infectious Diseases* 25(9): 1033–1043. https://doi.org/10.1016/S1473-3099(25)00118-5

11. Pendleton, Jack N., Sean P Gorman, and Brendan F Gilmore. 2013. “Clinical relevance of the ESKAPE pathogens.” *Expert Review of Anti-infective Therapy* 11(3): 297–308. https://doi.org/10.1586/eri.13.12

12. Shen, Wei, Shuai Le, Yan Li, and Fuquan Hu. 2016. “SeqKit: A cross-platform and ultrafast toolkit for FASTA/Q file manipulation.” *PLoS One* 11(10): e0163962. https://doi.org/10.1371/journal.pone.0163962

13. De Coster, Wouter, and Rosa Rademakers. 2023. “NanoPack2: population-scale evaluation of long-read sequencing data.” *Bioinformatics* 39(5): btad311. <https://doi.org/10.1093/bioinformatics/btad311>

14. Arango-Argoty, Gustavo A., Dongjuan Dai, Amy Pruden, Peter Vikesland, Lenwood S Heath, and Liqing Zhang. 2019. “NanoARG: a web service for detecting and contextualizing antimicrobial resistance genes from nanopore-derived metagenomes.” *Microbiome* 7(1): 88. https://doi.org/10.1186/s40168-019-0703-9

15. Rolbiecki, Damian, Łukasz Paukszto, Katarzyna Krawczyk, Ewa Korzeniewska, Jakub Sawicki, and Monika Harnisz. 2023. “Chlorine disinfection modifies the microbiome, resistome and mobilome of hospital wastewater - a nanopore long-read metagenomic approach.” *Journal of Hazardous Materials* 459: 132298. https://doi.org/10.1016/j.jhazmat.2023.132298

**Figure S1 Analysis of antibiotic resistome dynamics in hospital wastewater following disinfection using L-EasyARG.** (A) Stacked bar plot of ARG composition across samples pre- and post-disinfection. (B) PCoA illustrating the significant shift in the overall ARG profiles after disinfection. (C) Increased abundance of ARGs co-occurring with MGEs post-disinfection. (D) The abundance of plasmid-located ARGs marginally declined after disinfection relative to pre-disinfection controls, with no significant difference observed (*p = 0.346*). (E) Sankey diagram illustrating the distribution of the top 15 most abundant ARG subtypes (based on total abundance across all samples) between chromosomal and plasmid in pre- and post-disinfection samples. (F) Relative abundance of the top 20 ARG subtypes in HBPs pre- and post-disinfection. (G) Heatmap showing correlations between the top 5 high-risk HBPs and top 15 ARG subtypes (selected by abundance). The overall significance of the correlation structure between the ARG and HBP profiles was assessed using a Mantel test.
